# The Monetary Valuation of Lifetime Health Improvement and Life Expectancy Gains in Turkey

**DOI:** 10.3390/ijerph14101151

**Published:** 2017-09-29

**Authors:** Shihomi Ara, Cem Tekeşin

**Affiliations:** 1 Department of Economics, Hacettepe University, Ankara 06800, Turkey; 2 International Agribusiness Group, LLC. 33533 West Twelve Mile, Suite 145, Farmington Hills, MI 48331, USA; ctekesin@iag-group.com

**Keywords:** the value of a life year (VOLY), the value of the healthier and longer life (VHLL), air pollution, income elasticity of WTP, Contingent Valuation Method (CVM), Turkey

## Abstract

The main objective of this study is to estimate the monetary value of the gains of healthy days and life expectancy due to the ambient air quality standard that came into effect in 2014 by estimating the country-specific value of a life year (VOLY) and the value of the healthier and longer life (VHLL) for Turkey. Contingent valuation method is adopted to reveal individuals’ willingness to pay for an improvement of health condition and the extension of their life expectancy by avoiding respiratory and lung related illnesses. VHLL is composed of two parts, WTP for an extension of one’s life years (VOLY) and for an increase in the number of healthy days throughout one’s life time (VHLL-VOLY). We found that close to 80% of WTP is allocated to the latter component of VHLL and only 20% is for VOLY mainly due to Islamic beliefs of the respondents. A total of 1314 observations are collected by face-to-face interviews from Afsin-Elbistan, Kutahya-Tavsanli and Ankara. The estimated VHLL and VOLY are [41,750 TL, 10,258 TL] with all the observations, [30,185 TL, 7132 TL] for Afsin-Elbistan, [31,718 TL, 7081 TL] for Kutahya-Tavsanli and [52,334 TL, 14,813 TL] for Ankara. The Inverse-U shaped relationship between Age and WTP is confirmed. The income elasticities of WTP is found to be close to 0.5 for all study areas while an Inverse-U shaped relationship between the household income and Income Elasticity of WTP is observed in the income group based analysis. Age and household income are the two prominent determinants of VHLL.

## 1. Introduction

A new ambient air quality standard has been in effect in Turkey from the beginning of year 2014. The standard level for the annual average of particulate matter with a diameter of less than 10 micrometers (PM_10_) was 150 $\mu g/m^{3}$ prior to this date and is currently in a transition period to 40 $\mu g/m^{3}$ which is expected to be met by January 2019. Similarly for sulfur dioxide (SO_2_), the annual average target level is currently 20 $\mu g/m^{3},$ while it was 150 $\mu g/m^{3}$ until the end of 2013. These kind of drastic improvements in environmental quality come with the costs which can be in general readily calculated, yet the necessary evaluations of benefits of such positive changes in the environmental quality are largely missing in Turkey, including the impacts to human health expressed in a monetized fashion. The estimation of country-specific values for benefit calculations as well as the establishment of monetary valuation methodologies in a developing country such as in Turkey is an urgent task for proper environmental policy evaluations. In addition, an estimation of the value of a life year (VOLY) in one of the Islamic countries using a contingent valuation method raised a serious methodological question since people perceive their life expectancy to be pre-determined and never be changed.

The main objective of this article is to estimate the monetary value of the better health condition (measured as the number of healthy days) and extended life years as a result of the reduction of health risk due to an improvement of air quality in Turkey. The value of a life year (VOLY) is a more desirable measure of premature death from air pollution compared to the value of a statistical life (VSL) when the impact of air pollution is chronic [1,2]. Since chronic health damages from air pollution cause the situation of “*a lot of people losing a small amount of life expectancy*” instead of “*a small number of people losing a large amount of life expectancy*” as in traffic accidents, VOLY is discussed to be more appropriate to measure the changes in premature mortality due to air pollution especially for the people in good health condition. The ExternE project concluded that their clear preference is VOLY, especially in the valuation of time delayed mortality where the dose-response function provides the way to calculate the loss of life years [2].

Since we decided to consider an extension of life in the end of one’s life as the result of an improvement of health condition throughout the life time especially for the case of chronic illnesses, our presentation of contingent valuation (CV) question was as illustrated in the figure in Appendix A. Hence our estimated willingness to pay (WTP) contains two components, (1) an extension of one’s life and (2) an increase in healthy days throughout one’s life time. In order to avoid confusion, our composite good hereinafter is called “Value of the Healthier and Longer Life” or “VHLL”. The first component is used to calculate VOLY, and the second component is the WTP for the increased healthy days by avoiding respiratory and lung related illnesses, which can be linked to the reduction of air pollution (we took the approach of aggregating the annual WTP through one’s lifetime using the necessary discounting and considered it as the total WTP for the increase in total number of healthy days throughout ones’ life span and the one-year extension of one’s life year. We also assumed the constant share rate between the healthier life and the extension of life years for the rest of the stages of one’s life time separately. The changes in the WTP allocations for the healthier life and the longer life also could be investigated). The decomposition of each component will be conducted after the estimation of VHLL based on the average share of WTP allocated to the life extension and ones’ healthier life by our respondents.

Important contributions of this study are to fill three major research gaps. First, comparing to VSL studies, the studies directly estimating VOLY are quite limited. This study is expected to provide insights to reveal the VSL-VOLY relationship [3]. Second, the studies conducted in low-and middle-income countries are still significantly fewer than the ones from high-income countries. Estimates for low- and middle-income countries are typically derived by adjusting estimates from high income countries. There are great research needs to obtain the country-specific estimates in order to improve our understanding of the preferences of those who are directly affected by the environmental pollutions. In addition, it is also necessary to validate the (un)adaptability of estimates from developed to developing world. Third, there is no study, to our knowledge, conducted to reveal VOLY using Contingent Valuation Method (CVM) in Islamic countries, where there is a strong belief on pre-determined destiny and “only God knows” principle (Ref. [4] conducted a VSL study in Kuwait, one of the Islamic countries. We also estimated VSL for Turkey as reported in [5] without encountering this problem since VSL is derived based on the probability-based hypothetical questions. However, for the estimation of VOLY, we have to mention “an extension of one’s life year” directly, and it causes the conflict). Considering the large share of Islamic population in the world (>20%), it is also important to examine the applicability of CVM on estimation of VOLY.

In addition to the estimation of VOLY and VHLL in Turkey, we attempt to provide an evidence from one of the low- and mid-income countries to the discussion of: (a) age-VOLY/VHLL relationship [6], (b) the validity of the assumption of the largely used income elasticity being one in benefit transfers and (c) the relationship between the income elasticity and income levels [7,8,9,10,11,12]. To the best of our knowledge, this is the first study to estimate VOLY, VHLL and income elasticity of VOLY and VHLL in Turkey. 

VOLY and VHLL estimations are conducted by using the data collected in six cities (Afsin (population: 84,244), Elbistan (139,046), Kutahya (0.56 million), Tavsanli (101,001) and Ankara (4.9 million) in three provinces (Kahramanmaras, Kutahya and Ankara) in Turkey using CVM. Study areas were selected based on their development rankings (Ankara: 2nd, Kutahya: 38th, Kahramanmaras: 60th out of 81 provinces) in Turkey to represent large, middle and small cities together with the existing conditions of air pollution. 

The remainder of the article is structured as follows: Section 2 reviews the literature of existing VOLY studies and the research on income elasticities of WTP, Section 3 provides the theoretical background of CVM and the description of our CVM study including data collection and the structure of the hypothetical question, followed by Section 4 with the descriptive statistics of our data, estimated results of models with three specifications, and the derived income elasticities of WTP. Section 5 and Section 6 are allocated for the discussion and the conclusion.

## 2. Literature Review

### 2.1. Existing VOLY Literature

Compared to the studies estimating the value of a statistical life (VSL), the number of VOLY studies is limited [13,14,15,16,17,18]. One of the earliest literature estimating WTP of an increased life expectancy is published by [13,14]. Their estimated WTP for an increase in the life expectancy by one-year conditioning on that the individual survives to the age of 75 is found to be in the range of $400 to $1500. These values are significantly lower than values found in the later years. Three representative projects, External Cost of Energy (ExternE) [2], New Energy Externalities Developments for Sustainability (NEEDS) [15,16], and the one conducted for the Department of Environment, Food and Rural Affairs in United Kingdom (DEFRA) provide VOLY estimates for European countries [17]. ExternE calculated environmental external costs from air pollution and global warming. The preferred VOLY estimates ranges from €25,000 to €150,000 with the central value of €50,000 depending on the presented scenario though the sample size was quite small [2]. The project by DEFRA empirically estimated the monetary values of the health benefits of reducing air pollution for the United Kingdom (UK) and derived the value of one year gain in life expectancy with normal health as £27,630 [17]. NEEDS derived the monetary values of mortality and morbidity risks from air pollution and estimated VOLY to be €41,000 for EU15 + Switzerland, €33,000 for New Member Countries of EU by using CVM conducted in nine European countries [15,16]. The income elasticities of VOLY derived ranges between 0.156 and 0.5570 for EU16 and new member countries for a 3 or 6-month extension of lives. The estimated WTP for 3 and 6 months were found to be insensitive to the scope and the ratio WTP (6_month)/WTP (3_month) was found to be around 1.30. In a more recent study, Ref. [19] found VOLY for Greece as €41,000 as the result of CVM with open-ended question. 

As [20] discusses, the gap between the intention of researchers asking CVM question for VOLY and the actual interpretation and understanding of the question presented as in [15,16,20] by respondents worth an investigation. Even if the researchers are intending to reveal just an extension of life expectancy, presenting the figure illustrating both the extension of life year at the end of their life time and a shift of “ability to survive” curve to up-right direction could result in varying interpretations by the respondents. It is possible that some give more weight on an improvement in “ability to survive” and others may be simply focusing on the extension of life years. The interpretation of “ability to survive” could also be ambiguous. Although the WTP derived by [15,16] is calculated as VOLY, it is possible that the estimated VOLY is reflecting some of the “implicit” values imposed by respondents in addition to life expectancy extensions.

### 2.2. Income Elasticity of WTP

In our study, we found the income elasticity of VHLL as less than one for all cases in our study, meaning that people perceive the suggested health improvement as necessary goods (the elasticity less than 1), instead of luxury goods (the elasticity greater than 1). While our finding provides some insights to the selection of the income elasticity for the international unit-value transfer practices, it is not appropriate to use the domestically found income elasticities directly for the transfers between the countries with very different income levels. However, our estimated VOLY (and VSL in [5]) can be used to test the validity of the selection of the income elasticity of VOLY (VSL) from international unit-value transfers. 

There are mainly three groups of studies estimating income elasticities of WTP which are relevant to our study. The first group is the study on WTP for (mortality) risk reduction in one country or in one region. Most of the studies estimate VSL and derive one or more income elasticities of WTP (VSL) for the specific study area. Some derive multiple elasticities for different income levels within a country [8,21] while others derive country-specific elasticities within multiple countries in a region (i.e., EU) [7]. The second group of study on income elasticity of WTP is for (mortality) risk reductions in international benefit transfer context. Such transfers are conducted between countries or regions using income elasticity of WTP using the $WTP_{S}=WTP_{P}{\left(\frac{Y_{p}}{Y_{S}}\right)}^{\beta }$ formula, where s and p stand for study (the site where the value is transferred to) and policy site (where the value is transferred from), respectively, *Y* is typically PPP-adjusted GDP per capita, and *β* is the income elasticity of WTP because *β* may be different across different income groups. Multiple studies confirm the higher income elasticity of WTP for the lower income countries/populations [9,10,12,22]. Ref. [9,12] for example conclude the use of the elasticity of VSL greater than 1.0 in lower income populations is appropriate. The relationship between income elasticity of VSL and income levels with domestic/regional [7,8] and international settings [9,10,12,22] are examined. Except for [7], these studies found the negative relationship between the income elasticity of VSL and the income levels.

The third group of studies estimate the income elasticity of WTP for environmental quality improvements [23,24,25,26,27,28]. While [29] found the unitary income elasticity of WTP for air quality improvements, other studies such as [24,25,26,27] report the elasticities less than 1. As for the relationship between the elasticity and the income levels, Ref. [23,24,28] found a positive relationship for water quality improvements. The theoretical foundation of the positive relationship between income elasticity of WTP and income level is provided by [30]. 

The most relevant study which estimated the income elasticity of VOLY is [15,16]. They found that the income elasticity of VOLY is between 0.38 and 0.69 for all samples including EU countries. The elasticity was estimated as 0.2 for EU15 countries while it was 0.5 for new member countries. Hence, they found the higher income elasticity of VOLY for the lower income countries. 

There are limited, yet growing number of studies estimating income elasticities of WTP for risk reductions in developing countries. The existing income elasticities of WTP estimated for developing countries are quite mixed [21,31,32]. Ref. [31] found the income elasticity of WTP for mortality risk reduction as 1.95 using a meta-analysis based on the studies conducted in developing countries. Ref. [32] found an income elasticity of VSL being 2.44 for Iran. Ref. [31,32] did not report the income elasticity of VSL—income levels relationship. On the other hand, Ref. [21] found the income elasticity of WTP to reduce mortality risk in the subsequent year to be between 0.06 and 0.2, and to reduce the risk of developing Chronic Bronchitis being between 0 and 0.15 depending on the locations in China. In summary, while the income elasticities of VSL or VOLY estimated in developed countries with one or more countries within the same region often report the elasticity being less than 1.0, the results from developing countries are quite mixed. On the other hand, the international benefit transfer studies often found the greater than unity level of income elasticity of VSL for developing countries. Our study is expected to provide a country-specific income elasticity of WTP for Turkey.

## 3. Methods

### 3.1. Conceptual Model of Contingent Valuation Method

Random utility model is used to analyze dichotomous CV responses. Suppose that respondent *j*’s indirect utility can be written as:

(1) $$u_{ij}=v_{ij}(y_{j},z_{j},q^{i})+\epsilon_{ij}$$

where $v_{ij}$ is the deterministic component, $\epsilon_{ij}$ is the stochastic part of the function which is not observable to the researcher, *i* = 0 is the status-quo, *i* = 1 is the state of the alternative choice, $y_{j}$ is the *j*’s income, $z_{j}$ is a vector of household characteristics, $q^{i}$ is the quality or quantity indicator of environment/health to be valued [33]. More specifically, the indirect utility functions for each state are expressed as $u_{1j}=v_{1j}(y_{j}-b_{j},z_{j},q^{1})+\epsilon_{1j}$ and $u_{0j}=v_{1j}(y_{j},z_{j},q^{0})+\epsilon_{0j}$ for *i* = 1 and 0, respectively. $b_{j}$ is the amount of bid to be paid by *j* for an improvement of environment/health condition. The difference in the deterministic part of indirect utility is defined as $∆v_{j}=v_{1j}(y_{j}-b_{j},z_{j},q^{1})-v_{0j}(y_{j},z_{j},q^{0}).$ We adopt double-bounded dichotomous choice format in which we ask a follow-up question with the higher bid value followed by “yes” answer and the lower bid after “no” to the first bid amount. The following log likelihood function is maximized to estimate the parameters.

(2) $$lnL=\overset{N}{\underset{j=1}{\sum }}\{yy_{j}ln\pi_{yy}+yn_{j}ln\pi_{yn}+ny_{j}ln\pi_{ny}+nn_{j}ln\pi_{nn}\}$$

where *yy_j_*, *yn_j_*, *ny_j_* and *nn_j_* are the dummy variables for the respondent *j*’s answer for yes-yes, yes-no, no-yes and no-no, respectively. For example, if *j*’s response is yes-no, *yy_j_* = *ny_j_* = *nn_j_* = 0 and *yn_j_* = 1. $\pi_{yy}$, $\pi_{yn}$, $\pi_{ny}$, and $\pi_{nn}$ are defined as $\pi_{yy}\left(b,b^{U}\right)=\mathrm{Pr}\left[b^{U}\leq WTP\right]=1-F\left(b^{U}\right)$, $\pi_{yn}\left(b,b^{U}\right)=\mathrm{Pr}\left[b\leq WTP\leq b^{U}\right]=F\left(b^{U}\right)-F\left(b\right)$, $\pi_{ny}\left(b,b^{L}\right)=\mathrm{Pr}\left[b^{L}\leq WTP\leq b\right]=F\left(b\right)-F\left(b^{L}\right)$, and $\pi_{nn}\left(b,b^{L}\right)=\mathrm{Pr}\left[b^{L}\geq WTP\right]=F\left(b^{L}\right)$, respectively, where b is the first bid amount, $b^{U}$ is the higher bid value and $b^{L}$ is the lower bid value of the follow-up question. *N* is the total number of observations. In our analysis, we defined *F*( ) as logistic CDF and used log-linear function for $∆v_{j}$. 

Median and mean WTP for the base model are calculated as

(3) $$Median\;WTP=exp\left[-\left(\frac{\beta_{const}}{\beta_{bid}}\right)\right]$$

(4) $$Mean\;WTP=\int_{0}^{max\_b}\frac{1}{1+e^{-\left(\beta_{const}+\beta_{bid}lnb\right)}}db$$

where max_*b* is the maximum bid amount, $\beta_{const}$ and $\beta_{bid}$ are the maximum likelihood (ML) estimates for the constant and the bid, respectively. For the full models, $\beta_{const}$ is re-defined as $\bar{z}\beta_{z}$ where $\bar{z}$ is the sample average values of individual characteristics and $\beta_{z}$ is the vector of maximum likelihood estimates of each relevant variable. 

### 3.2. Estimation Models

The following four models are estimated to analyze various aspects of VHLL in each study area. The definitions of all variables are listed in Table 1. Model 1 (Equation (5)) is the base model simply with CONSTANT and BID variable estimated separately for Half-Year and One-Year versions:

(5) $$V=\beta_{0}+\beta_{1}lnBID$$

Model 2 (Equation (6)) includes the individual characteristic as formulated below. We estimated this model using the pooled data containing both Half-Year and One-Year versions for each study area. AGE variable is scaled as $SCAGE=\left(AGE-\bar{AGE}\right)/sd_{AGE}$ in order to avoid high correlations between AGE and AGE-squared terms.

(6) $$\begin{array}{cc} V=\beta_{0}+\beta_{1}SCAGE & +\beta_{2}SCAGE^{2}+\beta_{3}GENDER+\beta_{4}\left(\frac{HHINC}{1000}\right)+\beta_{5}NCHILD\\ & +\beta_{6}UNIV+\beta_{7}SMOKER+\beta_{8}SPORT+\beta_{9}OWNRESP+\beta_{10}EMERG\\ & +\beta_{11}ELDER+\beta_{12}ONEYR+\beta_{13}lnBID \end{array}$$

BoxCox transformation of WTP function is used to test the appropriate functional form for the estimation of the income elasticity of WTP Model 3 (Equation (7)). The estimated result confirms that the transformation coefficient for WTP is statistically significant and very close to 1 while the coefficient is tested statistically insignificantly different from zero for HHINC. Hence, we used the semi-log form Model 3’ (Equation (8)) for the estimation of the elasticity. The dependent variable WTP is calculated by using the midpoint approach ($b^{L}$/2 for No-No answer, $b^{H}$ for Yes-Yes answer, ($b+b^{L}$)/2 for No-Yes answer and ($b+b^{H}$)/2 for Yes-No answer) and the elasticity is calculated using the estimated coefficients and the mean WTP values.

(7) $$\frac{WTP^{\lambda 0}-1}{\lambda_{0}}=\beta_{0}+\beta_{1}\frac{HHINC^{\lambda 1}-1}{\lambda_{1}}+\beta_{2}ONEYR$$

(8) $$WTP=\beta_{0}+\beta_{1}log\left(HHINC\right)+\beta_{2}ONEYR$$

The description of each variable and the bid structure of the hypothetical question are summarized in the Table 1 and Table 2. The proportions of accepting the first bid (the summation of percentages reported in the 6th and 7th columns in Table 2) represent the close-to-monotonic relationships, except for 1000-1500-800 (BID-BID_HIGH-BID_LOW) for Afsin-Elbistan and 1000-1500-800 for Ankara. The bid values vary across regions due to (a) differences in household income levels across study regions and (b) the results of pre-tests. 

### 3.3. Decomposition of VHLL and VOLY

As we discussed in Section 1, our WTP estimates are for both an extension of ones’ life and an increase in healthy days throughout one’s life time. In order to meaningfully compare our results with the existing studies and to use our estimates in future studies, a decomposition of VOLY from the composite VHLL estimate is necessary. The decomposition is done by using the self-reported allocation of WTP for various components, namely (a) the pre-set treatment cost of 200 TL per year, (b) avoiding potential income loss, (c) avoiding pain and discomfort, (d) an extension of life expectancy and (e) other. Treatment cost was set fixed at 200 TL because the treatment costs could be quite different in public and private hospitals. In this question, we are interested in the allocation of WTP for each component. We derive the sum of the percentages of (a), (b), (c) as WTP for the better health and the percentage allocated to (d) as WTP for an extension of life expectancy as calculated in Table 3.

It is quite interesting to observe the similarities of such budget allocations across three study areas. 76.44% to 79.90% of WTP is allocated to the better health condition while the share for an extension of life-year ranges form 15.78% to 21.64%. To our surprise, VOLY share is approximately one-fifth of VHLL. The most probable reason for the small share for the extension of life expectancy is the Islamic belief of the impossibility of changing ones’ life expectancy based on their behavior. If so, the estimated VOLY in this study should be considered as the lower-bound value. 

### 3.4. Survey Design

The survey consists of four sections. The first section asks respondents’ basic socio-demographic characteristics and health conditions of own and the family members, the second section is a contingent valuation (CVM) study on minor symptoms which is reported in [34], the third section is allocated for a CVM which is analyzed in this article, and the fourth section wraps up by asking the level of confidence and understanding of the hypothetical questions, monthly household income, other source of income and debt.

**Figure A1:**
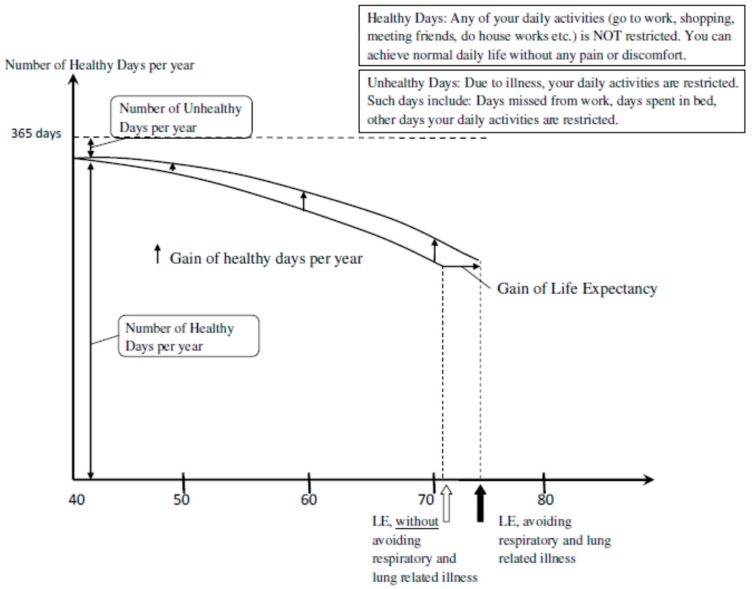
Life expectancy and healthy days.

The description of one (or half) year gain in respondent’s life expectancy (Table A1) as the result of better health condition starting today till the end of his/her life is visualized (Figure A1) and explained by using a figure prepared for each age group as in [15]. While the main characteristics of the figure is similar to the figure used in [15], some modifications have been made mainly in order to increase the comprehensibility of the question to our respondents. The major difference is the definition of the *y* axis while it is “ability to survive” in [15] and “number of healthy days per year” in our survey. Hence in our figure as shown in Appendix A, the vertical arrows represent the increases in the number of healthy days per year while the horizontal arrow describes the gain of life expectancy in the end of their expected life time. 

**Figure A2:**
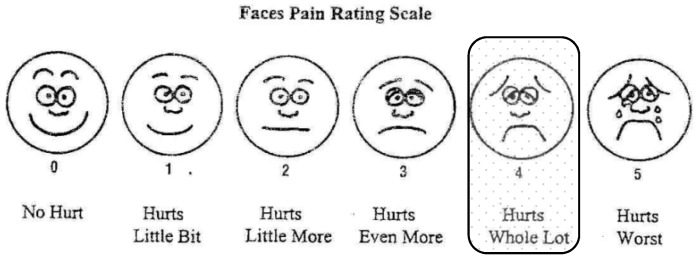
Level of pain.

We first explain the possible reasons for the cost for improving health condition and extending the life expectancy, then specify the improvement of health condition due to an avoidance of respiratory and lung related illnesses by paying a certain among of money every year for the rest of their lives. A detailed description of chronic bronchitis together with illustration of its level of discomfort (Figure A2) is provided together with the explanation of possible results due to the illness. We specified the illness as respiratory and lung related diseases in order to connect the illnesses to the level of air pollution. However, we did not provide the explicit linkage between the illness and air pollution to the respondents since people blame the government and claim that the government should pay for the damage, if the term “air pollution” is in the question. The following question is asked to reveal individuals’ WTP:

“*The avoidance of these illnesses will gain you a number of healthy days each year as you can see in Figure A1 and, you will have [Version 1: half a year more, Version 2: 1 more year] added to your average life expectancy in the end of your life. Suppose it will cost you 200 TL each year if you experience one of the respiratory illnesses stated earlier as an out-of-pocket treatment cost.*

*Would you pay [] TL every year for the rest of your life to avoid the respiratory and lung related illnesses entirely, gain the number of healthy days each year, and add [Version 1: half a year, Version 2: 1 more year] to your normal life expectancy? Please remember that if you agree to pay, you may have to give up some of the planned expenditure for the goods such as a good television, a smart phone or a computer.*”

The severity of the illness is fixed to “4: Hurts whole lot” in pain rating scale. Many Muslims believe in “already written destiny at birth” and they could not accept the idea of “extending life expectancy”. We asked a follow-up question for No-No answers and included it in our estimation if it is due to their budget constraints, but eliminated if the stated reasons are one of “*Scenarios seem to be unreal and I do not believe that the scenario will be realized. Hence, I did not consider the answers seriously* (Count = 51)”, “*I did not understand the scenario well. Therefore, I could not evaluate properly* (7)”, or a part of “*Others* (56)” answers. The majority who marked “Others” stated the reason as “Only God knows”. 

### 3.5. Data Collection

Prior to pre-tests in each location, multiple focus group discussions were held both in universities and local ministries in each location. Pre-tests were conducted in 21–25 September 2011 in Central Kutahya (*n* = 95) and Tavsanli (*n* = 85), 13–14 May 2012 in Ankara (*n* = 122) and 2 June 2012 in Elbistan (*n* = 119) by 10 trained interviewers under our direct supervision in each location. All subjects agreed to participate in this study were provided with clear objective and the nature of the study and understood its anonymity. The main survey for this version was conducted in 4–11 June in Afsin and Elbistan, 23 June–2 July in Central Kutahya and Tavsanli and 14–21 June 2012 in Ankara. The home addresses of random samples are obtained from the Turkish Statistical Institute for each location. Each interviewer visited the assigned houses and conducted face-to-face interviews. In case of absence, follow-up visits were made in the evenings. After omitting coding errors and protest votes, a total of 1314 samples, 514 observations for Afsin-Elbistan, 488 for Kutahya-Tavsanli and 312 for Ankara are included in our analysis. 

Given that the majority of population in Turkey is Muslim, two things are worth mentioning. First, it was quite surprising that the majority responded to the question seriously, regardless of their religious beliefs. However, the low share of WTP allocated to an extension of life expectancy indicates that the attention of respondents is given more to the healthier life years than the longer life. Second, 10–15% of respondents simply refused to answer/consider this type of question, mainly due to their religious beliefs. This indicates that our estimates do not include the data from a specific group of people with a certain characteristic (probably more “religious” people). Considering the fact that our interviewers reported that many of these “protest voters” accepted “better health” scenario, but not “longer life time”, we have to develop a method to use “better health” extensively and “extension of life” more implicitly, yet measure the monetary value of the LE extension. When we run the models with protest votes, we consistently find the lower mean and median WTP for all study areas and for both one and half year versions. This result indicates that if we have more “religious” population in our sample who refuse to consider the LE extension scenario, our VHLL and VOLY estimates will be lower. 

### 3.6. Discounting

Respondents are asked to reveal their WTP as the annual payments for the rest of their lives. Hence, it is critical to incorporate discounting into our VHLL and VOLY calculations. Although we attempted to estimate our sample-specific discount rate by asking Time-Money trade-off to each respondent (as 1. You can wait for one year and get 1100 TL at the end of the year. 2. You can receive 1000 TL now. Which one would you prefer?), we failed to do so for mainly two reasons. The first was the strong disbelief in earning “interests” due to Islamic belief by some respondents. The second reason was the prevailing myopic view and their distrust for the long-term financial commitments. Regardless of the reward offered for later reception of the money, many preferred receiving the money now than later, resulted in an unacceptably high estimate of discount rate. 

Instead, we decided to discount the bid amounts using annuity based on the remaining life years. The discounted bids were calculated as 1−1((1+i100)LIFEYR)i100×BID, where LIFEYR is the respondent-specific remaining life years derived as (Expected remaining life year based on his/her current age) minus (the current age of the respondent) and i is the discount rates varied between 0 and 10% (0%, 1%, 2%, 3%, 5%, 7% and 10%). Hence, our estimation models incorporate BID variable defined as the respondent-specific life-time payment. Therefore, the derived WTP is the estimated VHLL itself for the one-year version. VHLL is calculated as VOLY + VHLL* (the share of WTP for the healthier life) for the half-year version.

## 4. Results

### 4.1. Descriptive Statistics

In this section, we report the estimated results of Models 1, 2 and 3’. After eliminating coding errors, observations with critical missing values and protest votes, we used 514, 488 and 312 observations for Afsin-Elbistan, Kutahya-Tavsanli and Ankara, respectively. The descriptive statistics of the samples used for this analysis are listed in Table 4. The estimation of double-bounded dichotomous choice question was conducted by using the R program.

The monthly household income is approximately 1700 TL ($1123) for Afsin-Elbistan and Kutahya-Tavsanli and 2700 TL ($1781) for Ankara. According to the national census conducted in 2011, 715 TL, 1215 TL, 1726 TL, 2434 TL and 4983 TL are monthly household disposable income for the 1st through 5th quintiles. Women are under-represented in Afsin-Elbistan and Ankara, the average ages are around 40–42. The average numbers of children are the highest in Afsin-Elbistan (1.38) and the lowest in Ankara (0.75). While university graduates are 32% of respondents in Ankara, it is about 10% in other two areas. The national average is 10.3% for university and higher degree holders in 2011. Approximately half of the respondents exercise at least once a week while the rate is slightly lower for other two cities (approximately 40%). The smoking rate is high (40–48%) in all areas. Since the national average is 41.4% for male and 13.1% for female [35], our sample average is higher than the national average. The occurrences of respiratory diseases (Asthma, Chronic Bronchitis and Emphysema) is the highest in Afsin-Elbistan (35% of respondents), indicating the existence of high health risk factors in the area. The percentage of smokers are the highest in Ankara (48%) and the lowest in Afsin-Elbistan (40%). It is possible that the higher occurrences of respiratory diseases in Afsin-Elbistan caused the lower smoking rate in the area. 

One possible evidence of this hypothesis is the high percentage of those who have quitted smoking in Afsin-Elbistan (41%) compared to other cities (22% in Kutahya-Tavsanli and 19% in Ankara). Although the population descriptive statistics are not readily available for all the variables, Table 5 reports some of the key population variables for our study areas.

As one of other indicators of health issues in Afsin-Elbistan, only 38% of the respondents consider their health as “Good for their age (GOODHLTH)” while 46% and 51% of respondents from Kutahya-Tavsanli and Ankara consider their health as good. Similarly, 20% of the respondents perceive that their health condition is bad for their ages in Afsin-Elbistan while it is around 10% in other two areas. Together with the high occurrence of respiratory illnesses among respondents, family members of the respondents (wife or husband of the respondent (RESP_Partner), at least one of the children (RESP_Child), and respondents’ parents (RESP_Parent)) are also susceptible to respiratory diseases with 6% to 15% higher than other study areas. One of the reasons for this high health risk conditions in Afsin-Elbistan is the intensive use of low-quality coal for heating during winter due to lack of natural gas provision in the area. For non-respiratory illnesses such as cardio-vascular illnesses, cancer, diabetes, we did not observe any regional differences in their occurrences. The perceived air quality reveals the serious pollution in Afsin-Elbistan. 71% of respondents consider general air quality is either bad or very bad, and 96% perceive the air quality in winter is either bad or very bad in Afsin-Elbistan while the percentages are around 20% for all-year air quality and 46–61% for winter-time air quality considered bad or very bad in other study areas. While 45% answer that the air quality is getting worse in Afsin-Elbistan, 61% in Kutahya-Tavsanli where natural gas network was introduced in 2005 consider that the air quality is getting better. Considering the fact that 41% of Ankara respondents perceive that the air quality is getting worse and 46% consider the winter time air quality is bad or very bad although the three-year-average (2009–2011) of PM_10_ in Ankara is the lowest (64 μg/m^3^) among other study cities (100 μg/m^3^ for Afsin-Elbistan), the problem with air pollution, especially during winter is observed to persist and is needed to be solved with better policy instruments.

The comprehension of the hypothetical questions was measured by asking direct questions to the respondents. The question “Understanding the hypothesis questions is Very Difficult/Difficult/Easy/Very Easy” reveals that more than 86% (87% in Afsin-Elbistan, 91% in Kutahya-Tavsanli and 86% in Ankara) of respondents answered it was either “Easy” or “Very Easy” to understand the question and more than 92% (92% in Afsin-Elbistan, 94% in Kutahya-Tavsanli and 96% in Ankara) responded that they are either “Sure” or “Very Sure” of their answers for WTP questions.

### 4.2. Estimated Results of Base Model

Annual WTPs for the rest of their life time for a healthier and an extended life expectancy by half or one year are estimated based on the total sample sizes of 514 (Half: 256,One: 258), 488 (Half: 243, One: 245) and 312 (Half: 156, One: 156) for Afsin-Elbistan, Kutahya-Tavsanli and Ankara, respectively. In order to avoid anchoring effects, we used the same bid values for both half and one year versions. The estimated results based on the models with log-logistic, log-normal and Weibull distributions are compared and selected for each case based on the values of log-likelihood and Akaike Information Criteria (AIC) as reported in Table 6. All the estimated coefficients of the base model are statistically significant at one percent level with expected signs (Table 6). In Table 7, the estimated results using different discount rates are reported. For the half year version, we calculated VOLY as WTP × (the budget share for the 0.5 year extension of life years) × 2 and VHLL as VOLY + WTP × (the budget share for the healthier life years). For example, for Ankara using 1% discount rate and the half-year version, we estimated WTP as 33,108 TL. Since only 21.64% of this amount is allocated to a half-year extension of life years, we can derive VOLY for Ankara using 1% discount rate as 33,108 × 0.2164 × 2 = 14,329 TL. Since the rest of the WTP is allocated to the healthier life years, we do not multiply it by 2. 

As the result, VHLL is calculated as VOLY + WTP × (the share of the healthier life years), or VHLL = 14,329 + 33,108 × 0.781 = 66,216. The WTP shares used for the calculation is reported in Table 3. The last two columns of Table 7 present average values of VHLL and VOLY, simply averaged over VHLL and VOLY calculated based on the half and one year versions. VHLL is the lowest for Afsin-Elbistan (30,185 TL or 14,103 PPP-adjusted 2012 USD) and the highest for Ankara (52,344 TL or 34,576 USD) while VOLY is the lowest for Kutahya-Tavsanli (7081 TL or 4677 USD) and the highest for Ankara (14,813 TL or 9785 USD) using no discounting. The scope sensitivity can be calculated as VOLYone [12th column]/(VOLY based on half year result [7th column]/2) and reported in the last column of Table 7. It ranges between 1.54 (Kutahya-Tavsanli) and 1.71 (Ankara).

### 4.3. Estimated Result of a Model with Individual Characteristics

In order to identify the individual specific determinants of VHLL in each study area, we estimated the full model, Model 2 using the pooled data (half and one year versions). The result is listed in Table 8. 

The individual characteristics which statistically significantly influence WTP values are different across study locations. SCAGE, SCAGE^2^, HHNC and log(bid) variables are all estimated as statistically significant with the expected signs for all areas. We found that VHLLs are peaking at 26.9, 28.8 and 36.0 year-olds for ALL, Kutahya and Ankara, respectively. As for Afsin-Elbistan sample, we did not observe a peak within the range of age groups we included in our sample. When we analyze the relationship between AGE groups (18–24, 25–29, 30–34, …, 65–75) and the shares of WTP either to (1) the healthier life-year and (2) longer life, we found that the people in 65–75 age group are willing to allocate only 9% of their WTP to the longer life years while the share for other age groups are found as between 16 and 20 percent. It is possible that seniors simply shift their budget from the longer life expectancy to an increase in the healthier days. 

GENDER is negative significant only for Afsin-Elbistan, indicating the female respondents are willing to pay less. NCHILD is negative and significant for ALL and Afsin-Elbistan, meaning that those who have higher number of children are willing to pay less. The possible interpretation for the negative coefficient for NCHILD is the tighter budget constraint as the family grows. UNIV is positive and significant for ALL and Ankara, indicating that those who have graduated from a university is willing to pay more. SMOKER is positive and significant for Kutahya-Tavsanli, revealing that smokers are willing to pay more for VHLL. SPORT (exercise regularly) coefficients are positive and significant for ALL and Kutahya-Tavsanli samples. The probability of saying “yes” is higher for those who exercise regularly. OWNRESP (experienced respiratory disease) variable is found to have insignificant explanatory power. EMERG (visited emergency room due to respiratory diseases) was estimated as positive and significant only for ALL sample. ONEYR variable was positive and significant for ALL and Ankara samples, indicating that the version difference (half a year vs. one-year extension of life years) had statistically significant explanatory power only for Ankara. The highest share of the budget allocated for an extension of life expectancy (21.64%) compared to the other study areas could be one of the possible reasons for this significance in Ankara.

Based on the estimates from Model 2, marginal VHLLs (MVHLL) for each variable are calculated (Table 9). Marginal VHLLs are calculated as the difference between VHLL values based on state 0 and state 1 for the dummy variables (GENDER, UNIV, SMOKER, SPORT, OWNRESP, EMERG) and are derived as a difference in VHLL for one child and two children in the household for NCHILD variable. MVHLL for AGE is evaluated as a one-year increase in AGE from its mean value while it is measured as a 100 TL increase in HHINC from its mean value for HHINC variables. For all the difference calculation, we use the derived VHLL values based on the Krinsky and Robb’s method. Mean values are used for all other variables in the model. According to the MVHLL reported in Table 9, VHLLs decrease by 925, 587, 646 and 809 TL for ALL, Afsin-Elbistan, Kutahya-Tavsanli and Ankara samples, respectively, as a respondent get one year older from the mean ages of each region. When the monthly household income goes up by 100 TL from its current average income, VHLL is expected to go up by 954–1731 TL depending on the study area. For ALL sample results, an increase in the number of children from one to two results in a decrease in VHLL by 2657 TL while VHLL of those who are graduate from university, exercise regularly, have visited an emergency room are higher by 8514, 6210 and 12,673 TL, respectively. In Afsin-Elbistan, women are willing to pay less (−5900 TL). In Kutahya-Tavsanli, smokers are willing to pay greater (6701 TL) amount than non-smokers, while VHLL of those who exercise regularly is the higher by 8361 TL. In Ankara, the university graduates have the significantly higher VHLL (+24,479 TL) than non-graduates.

### 4.4. Income Elasticity of WTP

The semi-log specification (Model 3’) for the estimation of the income elasticity of WTP is used and the elasticity is derived as $\beta_{1}/\bar{WTP}$. According to the estimated result reported in Table 10, the elasticities are found to be 0.57, 0.50, 0.51 and 0.47 for ALL, Afsin-Elbistan, Kutahya-Tavsanli and Ankara, respectively. The resulting elasticities are close to the estimate (0.557) for the new member countries of EU (Czech Republic, Poland and Hungary) and the higher than the one for EU 16 (0.156) [15]. 

Within the context of income elasticity of VSL, while the elasticity is typically found in the range of 0.3 to 0.6 [36,37] for developed countries, the findings from developing countries are mixed and being in the range between 0.06 to 2.44 [21,31,32]. When we discuss the income elasticity of WTP for developing countries, we have to remind ourselves that WTP contains the meaning of both “willing to” and “cable of” payment. In fact, our interviewers reported the comments by respondents for the cases of “incapable of payment” although “willing to pay”. Among the respondents who answered No-No to the hypothetical questions, 75%, 76% and 63% of them stated that the reason is due to their tight budget constraint in Afsin-Elbistan, Kutahya-Tavsanli and Ankara, respectively. 

In order to further investigate the income and WTP relationship, Model 3’ is estimated for four different income groups, LOW (up to 999 TL), MID (1000–2499 TL), and HIGH (2500 and above) by using pooled version and study areas data (Table 10). When we calculate the income elasticity of WTP for each income group, it becomes clear that the elasticities vary for different income groups. The elasticities are estimated as 0.58, 0.72 and 0.62 for Low, Mid and High income groups, respectively. Hence, we observed an Inverse-U shaped relationship between income level and the income elastic of WTP. 

Based on the mean HHINC values for each group, 1% increase in the mean monthly income for the low-income group (763 TL to 771 TL), the mid-income group (1644 TL to 1660 TL) and the high-income group (3727 TL to 3764 TL) are expected to result in an increase in VHLL by 0.584% (12,448 TL to 12,520 TL), 0.726% (21,473 TL to 21,629 TL) and 0.624% (30,793 TL to 30,985 TL), respectively. 

The implications include (1) there is no significant difference in the elasticity among the study areas and it is around 0.5, (2) depending on the income groups, the elasticity could vary, and (3) our result suggests an Inverse-U shaped relationship between income level and the income elasticity of WTP and did not confirm the positive household income—the elasticity relationship as suggested by [7,23,24].

### 4.5. An Application for the Air Pollution Policy Evaluation

Based on the estimated results, the individual and total welfare gains in terms of health benefits are calculated in this section. The domestic standard for PM_10_ in Turkey is currently in transition from 150 $\mu g/m^{3}$ to 40 $\mu g/m^{3}$ by 2019. The three-year average (2009–2011) of PM_10_ levels are 100, 84 and 63 μg/m^3^ for Afsin-Elbistan, Kutahya-Tavsanli and Ankara, respectively. Hence, the expected reduction in PM_10_ levels by 2019 are 60, 43 and 24 in Afsin-Elbistan, Kutahya-Tavsanli and Ankara, respectively. In order to derive the welfare gains from the reduced years of life lost (YLL) due to PM_10_ reduction using our estimates, it is necessary to find the coefficient of exposure (PM_10_)—response (YLL) function which is suitable for our study setting. Most of such studies are conducted either in US [38,39,40,41,42]), Canada [43] or EU [44], and only a few studies have been conducted in developing countries [45,46]. The effects on life expectancy (LE) in years as the PM_2.5_ changes of 30 µg/m^3^ are summarized in [47]. The differences in LE varies between 1.1 and 5.4, and the average is 2.4. The change in LE from a study in [45] is 3.0, meaning when PM_2.5_ decreases by 30 µg/m^3^, the life expectancy increases by 3 years on average per person. Although the average coefficient is 2.4, since PM_10_ and PM_2.5_ levels are significantly higher in our study areas than the US, Canada or EU, we decided to adopt 3, slightly higher value than the average. Therefore, as the coefficient of exposure (PM_2.5_)-response (YLL), we adopt three reduced YLL per 30 µg/m^3^, or 0.1 YLL per 1 µg/m^3^.

Since YLL is derived based on fine particles with aerodynamic diameters equal to or less than 2.5 µm (PM_2.5_) instead of fine particles with aerodynamic diameters equal to or less than 10 µm (PM_10_), we have to convert PM_10_ into PM_2.5_ to be able to benefit from the existing studies. Since PM_2.5_/PM_10_ ratio was not found in our study areas, we relied on three studies for the value [48,49,50]. The first study is conducted in Greece. Since the ratio is derived from a medium sized city, Kozani (population: 70,000) with open-pit mine and lignite based electric power plants, the ratio is well represented for Afsin-Elbistan and Kutahya-Tavsanli where there also are open pit mines and lignite based power plants. The PM_2.5_/PM_10_ ratio found in the study is 0.42.

Ref. [49] found the ratio of 0.64 in Bursa, the fourth largest and an industrial city in Turkey. The study by [50] measured PM_2.5_ and PM_10_ for both indoor and outdoor in summer and winter in Kocaeli, one of the most industrialized and urbanized city with high population density in Turkey. The PM_2.5_/PM_10_ ratio they found are 0.65 (Indoor, Summer), 0.39 (Outdoor, Summer), 0.43 (Indoor, Winter) and 0.21 (Outdoor, Winter). The average over all four possible situations are 0.42, which coincide with the first study. We decided to use 0.42 for Afsin-Elbistan and 0.64 for Ankara. Combining exposure-response coefficient and PM_2.5_/PM_10_ ratio, for Afsin-Elbistan and Kutahya-Tavsanli, 1 µg/m^3^ PM_10_ causes a change in 0.042 YLL or 15.33 days, for Ankara, 1 µg/m^3^ PM_10_ causes a change in 0.064 YLL or 23.36 days. Given these assumptions, we can calculate welfare gains in terms of reduced years of life lost due to PM_10_ emission reduction to 2019 target level are derived for Afsin-Elbistan and Kutahya-Tavsanli as:

(9) $$\mathrm{Welfare}\;\mathrm{Gains}\;\left(\mathrm{TL}\right)=\left(0.042\;\mathrm{per}\;1\;\mu g/m^{2}\right)\times \mathrm{Emission}\;\mathrm{Reduction}\times \mathrm{VOLY}\times \mathrm{Population},\;\mathrm{and}\;\mathrm{for}\;\mathrm{Ankara}\;\;\mathrm{as}\mathrm{Welfare}\;\mathrm{Gains}\;\left(\mathrm{TL}\right)=\left(0.064\;\mathrm{per}\;1\;\mu g/m^{2}\right)\times \mathrm{Emission}\;\mathrm{Reduction}\times \mathrm{VOLY}\times \mathrm{Population}$$

The derived individual welfare gains of the PM_10_ reductions to the EU standard level by using the estimated VOLY are calculated as 17,973, 12,788 and 22,753 TL and once we aggregate for the population in each study area the total welfare gains in terms of the extended life years are derived as 3.97 billion, 3.94 billion and 91.20 billion TL for Afsin-Elbistan, Kutahya-Tavsanli and Ankara, respectively.

Welfare gain calculation can be made for the remaining part of VHLL, which corresponds to the WTP for the healthier life years. VHLL-VOLY are calculated as 31,492, 23,053, 24,053 and 37,531 TL for All, Afsin-Elbistan, Kutahya-Tavsanli and Ankara, respectively based on the values reported in the second last column in Table 7. These values are average individual welfare gains from avoiding respiratory and lung related illnesses. The city-wise welfare gains become 5.1 billion, 7.6 billion and 150 billion TL for Afsin-Elbistan, Kutahya-Tavsanli and Ankara, respectively. If we use the estimated Dose-Response coefficient for chronic bronchitis /100,000 which was derived by [51] as 61.2/100,000 per 10 $\mu g/m^{3}$ for PM_10_ and calculate the lifetime risk of chronic bronchitis given the simplest assumption of Poisson distribution for the occurrence of chronic bronchitis, the average lifetime risk avoided can be calculated as $1-e^{-\left(\frac{61.2}{10,0000}\right)\times 6\times 37}$ = 0.127 or 12.7% if we set the average lifetime PM_10_ dose as 60 and the average remaining life expectancy as 37 as in our sample. In order word, our respondents’ welfare gains from reducing the risk of chronic bronchitis by 12.7% is accounted partially in 31,492 TL lifetime payment on average. We have to admit, however, that this is a very preliminary calculation of the welfare gains from the health improvements and further research is necessary before actually being adopted to any policy evaluation.

## 5. Discussion

When policy makers use our derived VOLY in environmental policy evaluation and assessment, two cautions have to be taken. The first is that VHLL derived in this study includes both the value of increased healthy days throughout one’s life time and the value of an extension of the life expectancy by one year, and VOLY is calculated based on the share of WTP allocated for the latter cause. Our persuasion of more realistic and easily comprehensible scenario lead to the unique evaluation of two inseparable goods and resulted in somewhat unconventional evaluation of a composite good, VHLL. VOLY and VHLL values calculated using 0%, 1%, 2%, 3%, 5%, 7%, 10% discount rates are conveniently reported in Table 7. 

Regarding the scope sensitivity of VOLY, since only 15–20% of VHLL is allocated to VOLY and the remaining share is for the healthier life from now to the rest of the remaining life-years, the calculation using VHLL does not represent the intended scope sensitivity. Hence we derive the scope sensitivity using VOLY part of WTP and the VOLY(one)/VOLY(half) ratios are found to be between 1.54 and 1.71. The statistical (in)significance of ONEYR variable in our models does not provide us the evidence to the scope (in)sensitivity since we estimate VHLL, not VOLY directly from these models, although our above calculated ratios seem to be the higher than the ones found in the existing studies such as [15]. For example, Ref. [15] found the ratio of WTP (6 months)/WTP (3 months) as 1.25–1.32 for nine European countries. VOLY(6 months)/VOLY(3 months) ratios were found in the range between 1.10 (France) and 1.51 (Denmark) based on the median WTP. One of the possible reasons for the “low” scope sensitivity might be due to the unintentional expectation of “the healthier life-year” by the respondents in addition to an extension of one’s life-year by 3, 6 or 12 months in their studies.

As for Income Elasticity of WTP, our study provides one evidence of the elasticity being less than 1 for VHLL and it is somewhat consistent with our findings in our previous study that the income elasticities of the value of statistical life (VSL) ranging between 0.28 and 0.63 in [5]. Our result suggests that the assumption of the elasticity being 1.0 results in an overestimation of WTPs if $Y_{p}>Y_{s}$ while it will underestimate WTPs if $Y_{p}<Y_{s}.$

The preliminary calculation of welfare gains for the healthier life year part of VHLL (VHLL-VOLY) conducted in the end of Section 4.5 needs some clarification. In order to connect these welfare gains to air pollution policies, we need the information regarding to (1) the dose-response coefficients for the respiratory and lung related illnesses, including Chronic Bronchitis, Asthma and lower and upper respiratory illnesses and (2) the expected level of relevant pollutants (PM_10_, PM_2.5_, Ozone, SO_2_ and NO_2_) for the next 30–40 years in Turkey. Although our estimation of VOLY and the finding that VOLY share of VHLL is relatively low (approximately 20%) could be readily used in the policy evaluations, the estimates of (VHLL-VOLY) are preliminary and further research is necessary to be adopted to a policy assessment.

Lastly, as we discussed in Section 3.5, it is possible that we excluded a certain set of people (say, more religious people) from our analysis by eliminating “Only God Knows” protest votes which correspond approximately 7% of our total sample. Compared to the estimates of VOLY of €33,000 for new member countries reported in [15], our estimates are significantly lower. A careful approach is necessary to ask a hypothetical “life expectancy gains” questions in a country with large Muslim population since some people simply deny the idea of having a control over their life expectancy. If the share of this kind of “protestors” is high, we cannot use this type of CVM to reveal VOLY. For the future studies estimating VOLY in Islamic countries or targeting Muslim population, it will be better to decouple “an increase in the number of healthy days” and “an extension of life expectancy” and derive the value of the latter indirectly. 

## 6. Conclusions

In this study, Value of the Healthier and Longer Life (VHLL) and Value of a Life Year (VOLY) are estimated using face-to-face contingent valuation survey conducted in Turkey. In June–July 2012, 514, 488 and 312 questionnaires were collected in Afsin-Elbistan, Kutahya-Tavsanli and Ankara, respectively. Our study makes it possible to include the monetized health benefits in terms of improved and extended life years in cost-benefit analysis of environmental policies. It is expected to provide not only the country-specific WTP and welfare gains for Turkey but also the basis for the differences of evaluated welfares between developed and developing countries. 

Our preferred values of VHLL for All, Afsin-Elbistan, Kutahya-Tavsanli and Ankara are 41,750 TL ($27,578 in PPP-adjusted 2012 USD), 30,185 TL ($19,939), 31,718 TL ($20,952) and 52,344 TL ($34,576), respectively. As for VOLY, it is calculated as the share of the WTP allocated to an extension of life year (15.78–21.64%) as reported in Table 3 and derived as 10,258 TL ($6776), 7132 TL ($4705), 7081 TL ($4677) and 14,813 TL ($9785), respectively. The estimated VOLY using different discount rates are reported in Table 7. Our full model confirms the statistically significant effect of Age and Household Income on VHLL estimates for all regions. The scaled Age and Age-squared terms are found to be negative and significant, indicating the Inverse-U shaped relationship between Age and VHLL. Household income variable is positive and significant determinant of VHLL. Though the rest of the determinants vary from the study area to area, the negative gender effect (Afsin-Elbistan), the negative “the number of children” effect (Afsin-Elbistan), the positive university effect (Ankara), the positive “being a smoker” effect (Kutahya-Tavsanli) and the positive “exercising regularly” effect (Kutahya-Tavsanli) are confirmed in one or more study areas.

In order to estimate the income elasticity of WTP, we run the box-cox model and find that semi-log specification is appropriate for our data. Hence the elasticities are calculated using the mean WTPs and found to be 0.572, 0.501, 0.515 and 0.474 for All, Afsin-Elbistan, Kutahya-Tavsanli, and Ankara, respectively. We can safely conclude that the income elasticity of WTP (VHLL) is around 0.5 for our case. We observed an Inverse-U shaped relationship between the income elasticity of WTP and the income levels. The highest elasticity we found is for the middle income group (0.726) while the lowest was for the low income group (0.584). 

Based on the marginal VHLL estimates, we found that as age increases by 1 from its mean age, VHLL decreases by 587–925 TL depending on the region. Women are willing to pay 5900 TL less in Afsin-Elbsitan while we did not confirm the gender effect in other study areas. As monthly household income goes up by 100 TL from its mean level, VHLL increases by 954–1731 TL. An increase in the number of children from one to two is expected to result in a decreases in VHLL by 3366 TL in Afsin-Elbistan. University graduates are willing to pay 24,479 TL more in Ankara compared to the individuals without university degrees. Surprisingly, smokers are willing to pay more (by 6701 TL) in Kutahya-Tavsanli although no statistically meaningful relationship is confirmed for the other regions. The individual welfare gains in terms of VOLY by reducing PM_10_ to the EU air quality standard level are calculated as 17,973, 12,788 and 22,753 TL for Afsin-Elbistan, Kutahya-Tavsanli and Ankara, respectively.

## Figures and Tables

**Table 1:** Variable descriptions.

Variable	Description
BID	See Table 2 for the bid structure
AGE	Age of the respondent
SCAGE	Scaled AGE variable as (AGE-$\bar{AGE})/sd\left(AGE\right)$
GENDER	1 if the respondent is a female, 0 otherwise
HHINC/1000	Monthly household income/1000
NCHILD	Number of children under 18 in the household
UNIV	1 if having university or higher degree, 0 otherwise
SMOKER	1 if the respondent smokes regularly, 0 otherwise
SPORT	1 if the respondent exercises regularly (once a week or more), 0 otherwise
OWNRESP	1 if the respondent has experienced (experiencing) respiratory diseases including Asthma, Chronic Bronchitis, and Emphysema, 0 otherwise
EMERG	1 if the respondent visited emergency room due to respiratory and health diseases in last three years
ELDER	1 if the respondent has an experience of caring elderly people at home, 0 otherwise
ONEYR	1 if the survey version asks one year of life-year extension, 0 otherwise

**Table 2:** Bid Structure and response ratios.

Version	CITY	BID	BID HIGH	BID LOW	Yes-Yes	Yes-No	No-Yes	No-No	*n*
VOLY (Pooled)	Afsin-Elbistan	300	400	200	65 (52%)	15 (12%)	4 (3%)	39 (31%)	123
		400	600	300	56 (44%)	23 (18%)	6 (5%)	43 (34%)	128
		600	800	400	23 (20%)	14 (12%)	15 (13%)	65 (56%)	117
		800	1000	600	22 (21%)	3 (3%)	6 (6%)	72 (70%)	103
		1000	1500	800	19 (44%)	13 (30%)	0 (0%)	11 (26%)	43
	Kutahya-Tavsanli	200	400	100	60 (67%)	16 (18%)	4 (4%)	9 (10%)	89
		400	600	200	47 (49%)	13 (14%)	6 (6%)	30 (31%)	96
		600	800	400	24 (45%)	5 (9%)	4 (8%)	20 (38%)	53
		800	1000	600	34 (24%)	9 (6%)	11 (8%)	87 (62%)	141
		1000	1500	800	23 (23%)	9 (9%)	3 (3%)	63 (64%)	98
		1500	3000	1000	0 (0%)	2 (18%)	1 (9%)	8 (73%)	11
	Ankara	300	400	200	4 (100%)	0 (0%)	0 (0%)	0 (0%)	4
		400	600	300	36 (57%)	10 (16%)	6 (10%)	11 (17%)	63
		600	800	400	1 (2%)	22 (48%)	8 (17%)	15 (33%)	46
		800	1000	600	23 (32%)	2 (3%)	23(32%)	23 (32%)	71
		1000	1500	800	23 (56%)	17 (41%)	0 (0%)	1 (2%)	41
		1500	3000	1000	9 (14%)	9 (14%)	6 (9%)	41 (63%)	65
		3000	6000	1500	1 (5%)	4 (18%)	2 (9%)	15 (68%)	22

**Table 3:** The Allocation of WTP for different components.

		Mean	St. Dev.	Min	Max
Afsin-Elbistan	Avoiding Treatment Cost (200 TL)	38.99%	16%	13%	100%
	Avoiding Loss in Income	5.48%	11%	0%	67%
	Avoiding Pain/Discomfort	35.43%	23%	0%	87%
	Better Health Condition *	79.90%	23%	13%	100%
	Extension of Life Expectancy **	17.11%	21%	0%	87%
	Others	0.58%	4%	0%	45%
Kutahya-Tavsanli	Avoiding Treatment Cost (200 TL)	38.47%	22%	13%	100%
	Avoiding Loss in Income	6.81%	13%	0%	75%
	Avoiding Pain/Discomfort	32.31%	26%	0%	87%
	Better Health Condition	77.97%	24%	13%	100%
	Extension of Life Expectancy	15.78%	22%	0%	87%
	Others	0.29%	3%	0%	40%
Ankara	Avoiding Treatment Cost (200 TL)	27.82%	14%	3%	67%
	Avoiding Loss in Income	5.54%	12%	0%	52%
	Avoiding Pain/Discomfort	43.08%	30%	0%	97%
	Better Health Condition	76.44%	27%	7%	100%
	Extension of Life Expectancy	21.64%	27%	0%	93%
	Others	0.00%	0%	0%	0%

* Used as the share for the better health in our calculation. ** Used as the share for the extended life-year in our calculation.

**Table 4:** Descriptive statistics of variables.

Variable	ALL		Afsin-Elbistan		Kutahya-Tavsanli		Ankara	
	Mean	Std. Dev.	Mean	Std. Dev.	Mean	Std. Dev.	Mean	Std. Dev.
AGE	41.64	12.83	40.25	12.48	42.6	13.18	42.45	12.67
GENDER	0.45	0.5	0.39	0.49	0.51	0.5	0.44	0.5
HHINC	1962.42	1297.54	1701.47	1245.37	1768.57	1020.57	2695.51	1485.92
NCHILD	1.05	1.09	1.38	1.22	0.89	0.94	0.75	0.92
UNIV	0.16	0.38	0.11	0.31	0.1	0.34	0.32	0.49
SMOKER	0.43	0.51	0.4	0.49	0.42	0.51	0.48	0.56
SPORT	0.42	0.49	0.41	0.49	0.38	0.49	0.49	0.5
OWNRESP	0.3	0.46	0.35	0.48	0.28	0.45	0.26	0.44
EMERG	0.06	0.23	0.08	0.27	0.05	0.21	0.04	0.2
ELDER	0.29	0.46	0.32	0.47	0.27	0.45	0.28	0.45
ONEYR	0.5	0.5	0.5	0.5	0.5	0.5	0.5	0.5
GOODHLTH	0.44	0.5	0.38	0.49	0.46	0.5	0.51	0.5
BADHLTH	0.14	0.35	0.2	0.4	0.09	0.29	0.11	0.32
RESP_Partner	0.18	0.38	0.22	0.42	0.16	0.36	0.14	0.35
RESP_Child	0.2	0.4	0.24	0.42	0.17	0.37	0.18	0.39
RESP_Parents	0.24	0.43	0.32	0.47	0.17	0.37	0.22	0.42
AQ_BAD	0.39	0.49	0.71	0.45	0.18	0.38	0.21	0.4
AQ_BAD_Winter	0.71	0.45	0.96	0.21	0.61	0.49	0.46	0.5
AQ_BETTER	0.32	0.47	0.04	0.18	0.61	0.49	0.31	0.46
AQ_WORSE	0.35	0.48	0.45	0.5	0.2	0.4	0.41	0.49

**Table 5:** Population statistics.

	Afsin-Elbistan	Kutahya-Tavsanli	Ankara	
GENDER				
Gender (% Female)	49%	50%	50%	
AGE				
AGE 0–14	30.7%	19.0%	21.8%	
AGE 15–19	9.35%	7.88%	7.56%	
AGE 20–24	7.6%	9.1%	8.5%	
AGE 25–29	8.2%	7.6%	8.8%	
AGE 30–34	8.4%	7.7%	9.1%	
AGE 35–39	7.0%	7.3%	8.2%	
AGE 40–44	6.0%	6.7%	7.5%	
AGE 45–49	5.2%	6.7%	7.0%	
AGE 50–54	4.2%	6.6%	6.0%	
AGE 55–59	3.8%	5.8%	4.9%	
AGE 60–64	2.9%	4.8%	3.6%	
AGE 65–74	4.1%	6.5%	4.3%	
AGE 75+	2.6%	4.2%	2.7%	
EDUCATION				
University+	7.7%	8.5%	19.5%	
High School	18.4%	21.8%	27.1%	
Middle School	4.6%	4.5%	6.2%	
Elementary School	50.7%	53.5%	37.4%	
Not completed Elementary School	7.2%	7.5%	2.9%	
Illiterate	8.2%	3.5%	2.9%	
MONTHLY HOUSEHOLD DISPOSABLE INCOME (2014)				
	TR63 (including Afsin-Elbistan)	TR33 (including Kutahya)	Ankara	Turkey
Mean	1854	2398	3491	2667
Median	1410	1790	2671	2076

**Table 6:** Estimated results of base model.

Sample	ALL				Afsin-Elbistan				Kutahya-Tavsanli				Ankara			
LE Extension	Half		One		Half		One		Half		One		Half		One	
Distribution	Weibull		Weibull		Log-L		Log-N		Weibull		Weibull		Log-L		Log-N	
Constant	7.189	***	7.609	***	9.877	***	5.505	***	6.087	***	6.873	***	13.788	***	9.858	***
	(0.481)		(0.483)		(1.115)		(0.711)		(0.823)		(0.803)		(1.674)		(0.928)	
log(BID)	−0.700	***	−0.724	***	−1.031	***	−0.567	***	−0.590	***	−0.660	***	−1.379	***	−0.944	***
	(0.047)		(0.047)		(0.113)		(0.072)		(0.081)		(0.078)		(0.164)		(0.089)	
*n*	655		659		256		258		243		245		156		156	
WTP (Median)	17,097		22,097		14,484		16,525		16,342		19,011		22,035		34,184	
WTP (Mean, truncated at the maximum bid)	34,378		44,158		26,778		29,809		29,085		31,580		40,125		56,651	
95% LCI for mean WTP	30,399		37,858		23,344		26,010		24,830		27,163		33,065		46,094	
95% UCI for mean WTP	39,657		53,284		30,560		34,694		33,357		36,369		49,773		71,646	

*** corresponds to statistically significance levels of 10%, 5% and 1%, respectively. Mean WTPs are calculated by truncating at the maximum bids (1500, 3000 and 6000 TL for Afsin−Elbistan, Kutahya−Tavsanli and Ankara, respectively). WTP in TL can be converted into WTP in PPP adjusted 2012 USD as VOLY(TL) × 1.189/1.8.

**Table 7:** Estimated results of discounted VHLL and VOLY.

		Half Year Version Based					One Year Version Based					Average		Scope ****
		Dist.	VHLL ***	95% LCI	95% UCI	VOLY **	Dist.	VHLL	95% LCI	95% UCI	VOLY *	VHLL	VOLY	VOLYone/VOLYhalf
No Discount	ALL	Weibull	39,342	34,789	45,328	12,493	Weibull	44,158	37,858	53,284	8023	41,750	10,258	1.64
	Afsin	Log-L	30,559	26,640	34,875	9164	Log-N	29,810	26,010	34,694	5100	30,185	7132	1.56
	Kutahya	Weibull	31,857	27,196	36,536	9179	Weibull	31,580	27,163	36,369	4983	31,718	7081	1.54
	Ankara	Log-L	48,038	39,585	59,588	17,366	Log-N	56,651	46,094	71,646	12,259	52,344	14,813	1.71
1% Discount	ALL	Weibull	31,980	28,048	36,588	10,155	Weibull	35,319	30,024	42,124	6418	33,650	8286	1.63
	Afsin	Log-L	24,308	21,026	27,618	7289	Log-N	23,677	20,595	26,828	4051	23,992	5670	1.56
	Kutahya	Weibull	25,321	21,623	28,917	7296	Weibull	25,418	21,776	29,284	4011	25,369	5653	1.55
	Ankara	Log-L	39,637	32,789	49,416	14,329	Log-N	45,479	37,373	57,683	9842	42,558	12,085	1.69
2% Discount	ALL	Weibull	26,468	23,246	30,600	8405	Weibull	28,939	25,012	34,205	5258	27,703	6831	1.63
	Afsin	Log-L	19,767	17,201	22,565	5927	Log-N	19,236	16,878	21,890	3291	19,501	4609	1.56
	Kutahya	Weibull	20,588	17,965	23,676	5932	Weibull	21,656	18,687	25,008	3417	21,122	4675	1.58
	Ankara	Log-L	33,206	27,453	40,482	12,004	Log-N	37,358	30,658	46,911	8084	35,282	10,044	1.67
3% Discount	ALL	Weibull	22,282	19,432	25,817	7076	Weibull	24,216	20,858	28,289	4400	23,249	5738	1.62
	Afsin	Log-L	16,401	14,494	18,686	4918	Log-L	15,933	13,987	17,942	2726	16,167	3822	1.55
	Kutahya	Weibull	17,085	14,812	19,281	4923	Weibull	18,650	15,787	21,692	2943	17,867	3933	1.60
	Ankara	Log-L	28,234	23,438	35,363	10,207	Log-N	31,313	25,811	38,928	6776	29,773	8491	1.66
5% Discount	ALL	Weibull	16,529	14,377	19,300	5249	Weibull	17,876	15,572	20,767	3248	17,203	4248	1.62
	Afsin	Log-L	11,901	10,605	13,414	3569	Log-L	11,546	10,221	13,107	1976	11,724	2772	1.55
	Kutahya	Weibull	12,413	10,811	14,069	3577	Weibull	14,273	12,090	16,728	2252	13,343	2915	1.63
	Ankara	Log-L	21,254	17,714	26,231	7683	Log-N	23,151	18,997	28,229	5010	22,202	6347	1.65
7% Discount	ALL	Weibull	12,894	11,311	14,970	4095	Weibull	13,943	12,263	16,397	2533	13,419	3314	1.62
	Afsin	Log-L	9149	8176	10,282	2743	Log-L	8873	7850	9920	1518	9011	2131	1.55
	Kutahya	Weibull	9558	8341	10,739	2754	Weibull	11,348	9687	13,198	1791	10,453	2272	1.65
	Ankara	Log-L	16,748	13,800	21,213	6055	Log-N	18,060	15,106	21,790	3908	17,404	4981	1.65
10% Discount	ALL	Log-L	11,410	17,676	22,784	3623	Weibull	10,335	9052	12,033	1878	10,873	2751	1.52
	Afsin	Log-L	7397	10,520	13,106	2218	Log-L	6482	5777	7237	1109	6939	1663	1.50
	Kutahya	Weibull	6994	11,265	14,260	2015	Weibull	8527	7261	10,137	1346	7760	1680	1.67
	Ankara	Log-L	12,505	10,333	15,932	4521	Log-N	13,382	11,081	16,236	2896	12,944	3708	1.64

* VOLYs are derived as 18.17%, 17.11%, 15.78% and 21.64% of VHLL for ALL, Afsin-Elbistan, Kutahya and Ankara, respectively. ** VOLY based on 0.5 year version is calculated as WTP × (the share for VOLY) × 2. *** VHLL for 0.5 year version is calculated as VOLY + WTP × (the share for the value for the healthier life: 0.781, 0.799, 0.7797 and 0.7644 for ALL, Afsin-Elbistan, Kutahya and Ankara, respectively.) **** The ratio between VOLYone and VOLYhalf is calculated as (the one-year based VOLY)/(the half-year based VOLY/2).

**Table 8:** Estimated results for Model 2.

Sample	ALL		Afsin-Elbistan		Kutahya-Tavsanli		Ankara	
Distribution	Weibull		Log-L		Weibull		Log-L	
Constant	9.430	***	13.339	***	7.893	***	19.963	***
	(0.466)		(1.204)		(0.819)		(1.691)	
SCAGE	−0.344	***	−0.551	***	−0.323	***	−0.441	***
	(0.043)		(0.113)		(0.075)		(0.143)	
SQSCAGE	−0.150	***	−0.148	**	−0.155	**	−0.453	***
	(0.035)		(0.084)		(0.060)		(0.139)	
GENDER	−0.083		−0.458	**	0.236		0.187	
	(0.079)		(0.205)		(0.148)		(0.244)	
HHINC/1000	0.499	***	0.721	***	0.653	***	0.552	***
	(0.043)		(0.105)		(0.089)		(0.091)	
NCHILD	−0.081	**	−0.260	***	0.073		−0.063	
	(0.038)		(0.084)		(0.075)		(0.137)	
UNIV	0.232	*	0.001		0.317		1.040	***
	(0.122)		(0.325)		(0.251)		(0.291)	
SMOKER	0.124		0.103		0.262	*	0.053	
	(0.077)		(0.205)		(0.146)		(0.211)	
SPORT	0.181	**	0.142		0.326	**	0.017	
	(0.077)		(0.192)		(0.137)		(0.243)	
OWNRESP	−0.020		0.006		−0.095		0.256	
	(0.081)		(0.199)		(0.143)		(0.278)	
EMERG	0.325	**	0.424		0.276		0.469	
	(0.153)		(0.342)		(0.258)		(0.622)	
ELDER	0.090		0.156		0.217		−0.243	
	(0.080)		(0.199)		(0.142)		(0.275)	
ONEYR	0.232	***	0.037		0.167		0.922	***
	(0.073)		(0.183)		(0.124)		(0.241)	
log(bid)	−1.015	***	−1.450	***	−0.922	***	−2.135	***
	(0.045)		(0.121)		(0.080)		(0.166)	
*N*	1314		514		488		312	
LogL	−1382.8		−529.6		−453.4		−362.4	
WTP (Median)	20,784		16,476		19,567		28,210	
**WTP (truncated at the maximum bid)**	**29,641**		**25,505**		**27,984**		**40,132**	
95% LCI mean WTP	27,342		23,042		24,751		35,352	
95% UCI mean WTP	32,284		28,448		31,218		46,915	

*, **, *** corresponds to statistically significance levels of 10%, 5% and 1%, respectively. Mean WTPs are calculated by truncating at the maximum bids (1500, 3000 and 6000 TL for Afsin−Elbistan, Kutahya−Tavsanli and Ankara, respectively). Median WTPs are calculated with average values of each independent variable.

**Table 9:** Marginal VHLL based on Model 2.

Marginal VHLL (TL)					
	Description	ALL	Afsin-Elbistan	Kutahya-Tavsanli	Ankara
AGE	mean(AGE) ≥ mean(AGE) + 1	**−925**	**−587**	**−646**	**−809**
GENDER	Women (Gender = 0 ≥ 1)	−2791	**−5900**	6002	4067
HHINC	mean(HHINC) ≥ mean(HHINC) + 100 TL	**1731**	**954**	**1681**	**1209**
NCHILD	1 ≥ 2 kids	**−2657**	**−3366**	1883	−1343
UNIV	University Graduates (UNIV = 0 ≥ 1)	**8514**	14	8261	**24,479**
SMOKER	Smoker (SMOKER = 0 ≥ 1)	4219	1355	**6701**	1149
SPORT	Exercise Regularly (SPORT = 0 ≥ 1)	**6210**	1866	**8361**	363
OWNRESP	Experienced Respiratory Illnesses (OWNRESP = 0 ≥ 1)	−689	82	−2424	5699
EMERG	Visited Emergency Room (EMERG = 0 ≥ 1)	**12,673**	5821	7217	11,090
ELDER	Watched a Senior at Home (ELDER = 0 ≥ 1)	3098	2059	5595	−5155
ONEYR	One Year Version (ONEYR = 0 ≥ 1)	**7856**	483	4270	**20,058**

Bold values are based on the estimated coefficients that are statistically significant at least at 10% levels.

**Table 10:** Estimated results for income elasticity model (Model 3’).

Sample	Dependent Variable: WTP *													
	ALL		Afsin-Elbistan		Kutahya-Tavsanli		Ankara		LOW (<1000 TL)		MID (1000–2499 TL)		HIGH (>2500 TL)	
Constant	−70,352	***	−45,480	***	−54,266	***	−88,060	***	−35,998	*	−95,019	***	−126,906	***
	(6118)		(6397)		(8763)		(21,746)		(21,274)		(21,606)		(37,395)	
log(HHINC)	12,324	***	8780	***	9966	***	14,968	***	7273	**	15,593	***	19,223	***
	(824)		(885)		(1188)		(2786)		(3217)		(2926)		(4563)	
ONEYR	1682	*	−861		1094		7502	**	589		2943	**	820	
	(1020)		(1121)		(1270)		(3222)		(1191)		(1494)		(2530)	
*n*	1314		514		488		312		350		615		349	
AIC	29,554		11,175		10,710		7290		7517		13,837		8024.5	
MEAN(WTP †)	21,544		17,534		19,355		31,575		12,448		21,473		30,793	
Income Elasticity of Demand ‡	0.572		0.501		0.515		0.474		0.584		0.726		0.624	

† WTP is derived by using the midpoint approach (WTP for NN answer = BIDL/2, for YY = BIDH, for NY = (BID1 + BIDL)/2 and for YN = (BID1 + BIDH)/2. ‡ Income Elasticity of Demand is calculated as $\beta_{\log\left(HHINC\right)}/\bar{WTP}$. *, **, *** corresponds to statistically significance levels of 10%, 5% and 1%, respectively.

## Appendix A. Hypothetical Question for VOLY

Let’s now consider the effect of the health condition on your life expectancy. Life expectancy is the number of years you can expect to live, depending on how old you are now. For example, a baby girl born in 2006 has a life expectancy of 75 years, and a baby boy has a life expectancy of 71 years.

**Table A1:** Life expectancy in Turkey.

Age	Men	Women
Birth	71	75
20–24	53	58
25–29	49	53
30–34	44	48
35–39	39	43
40–44	34	38
45–49	30	34
50–54	26	29
55–59	21	25
60–64	18	20
65–69	14	16
70–74	11	13
75–79	8	9

For someone of your age, you can expect to live another ________ years on average. (For example, if you are a 20-year-old man, you will expect to live another 53 years on average.) Improved health condition today and for the remaining of your life time could bring you a certain gain in life expectancy since better health status will slow down your aging process.

In order to increase the number of healthy days each year and decrease the risk of experiencing a disease like chronic bronchitis which could even cause a death, there are certain things you can do. However, those measures usually require your effort and payment. For example, going to a hospital regularly every year for health check, staying indoor when air pollution is severe, staying away from people who smoke, quitting smoking if you smoke, switching from coal-based heating stove to central heating system, purchasing air-purifier at home, and checking indoor mold and dust at home regularly are among the things you can do for your healthier and longer life.

Suppose if you pay a certain amount of money every year for the rest of your life, you can avoid the occurrence of respiratory and lung related illnesses in your life. The avoided illnesses include chronic bronchitis, asthma, upper and lower respiratory infections. For example, the major symptoms of chronic bronchitis include: (i) intensive coughing that lasts for 3 months or more per year, (ii) wheezing, (iii) shortness of breath, (iv) production of sputum with yellow or green color with small amount of blood. These symptoms are caused by the irritation to the respiratory epithelium of the bronchi. Chronic bronchitis may cause you to be hospitalized, visit emergency room or doctors. You may need to take days off from your work and it may cause you to lose some part of your income. When the symptom is severe, your daily activities are also restricted. Imagine the level of pain and discomfort is “4” in the pain scale below.

The avoidance of these illnesses will gain you a number of healthy days each year as you can see in Figure A1 and, you will have [Version 1: half a year more, Version 2: 1 more year] added to your average life expectancy in the end of your life. Suppose it will cost you 200 TL each year if you experience one of the respiratory illnesses stated earlier as an out-of-pocket treatment cost.

Q1. How do you evaluate the gains of healthy days each year and extended life expectancy

*1. Significantly positively*	*2. Positively*	*3. Neutral*
*4. Negatively*	*5. Significantly negatively*	*6. Don’t know*

Q2. Would you pay [] TL every year for the rest of your life to avoid the respiratory and lung related illnesses entirely, gain the number of healthy days each year, and add [Version 1: half a year, Version 2: 1 more year] to your normal life expectancy? Please remember that if you agree to pay, you may have to give up some of the planned expenditure for the goods such as a good television, a smart phone or a computer.

*1. Yes*	*2. No*	*3. Don’t know*
